# Polyamines: Functions, Metabolism, and Role in Human Disease Management

**DOI:** 10.3390/medsci9020044

**Published:** 2021-06-09

**Authors:** Narashans Alok Sagar, Swarnava Tarafdar, Surbhi Agarwal, Ayon Tarafdar, Sunil Sharma

**Affiliations:** 1 Department of Agriculture and Environmental Sciences, National Institute of Food Technology Entrepreneurship and Management, Kundli, Sonepat 131028, Haryana, India; 2 Food Microbiology Lab, Division of Livestock Products Technology, ICAR-Indian Veterinary Research Institute, Izatnagar 243122, Uttar Pradesh, India; 3 Department of Radiodiagnosis and Imaging, All India Institute of Medical Science, Rishikesh 249203, Uttarakhand, India; dr.tarafdarswarnava@gmail.com; 4 Department of Hematology, Post Graduate Institute of Medical Education and Research, Chandigarh 160012, India; surbhiagarwal171990@gmail.com; 5 Livestock Production and Management Section, ICAR-Indian Veterinary Research Institute, Izatnagar 243122, Uttar Pradesh, India; ayontarafdar@gmail.com

**Keywords:** polyamines, biosynthesis, nutritional role, human health, disease prevention

## Abstract

Putrescine, spermine, and spermidine are the important polyamines (PAs), found in all living organisms. PAs are formed by the decarboxylation of amino acids, and they facilitate cell growth and development via different cellular responses. PAs are the integrated part of the cellular and genetic metabolism and help in transcription, translation, signaling, and post-translational modifications. At the cellular level, PA concentration may influence the condition of various diseases in the body. For instance, a high PA level is detrimental to patients suffering from aging, cognitive impairment, and cancer. The levels of PAs decline with age in humans, which is associated with different health disorders. On the other hand, PAs reduce the risk of many cardiovascular diseases and increase longevity, when taken in an optimum quantity. Therefore, a controlled diet is an easy way to maintain the level of PAs in the body. Based on the nutritional intake of PAs, healthy cell functioning can be maintained. Moreover, several diseases can also be controlled to a higher extend via maintaining the metabolism of PAs. The present review discusses the types, important functions, and metabolism of PAs in humans. It also highlights the nutritional role of PAs in the prevention of various diseases.

## 1. Introduction

Polyamines (PAs), such as putrescine (PUT), spermine (SPE), and spermidine (SPD), are organic polycationic alkylamines, which are synthesized from L-ornithine or by the decarboxylation of amino acids [1,2,3]. They are found in all living cells and mammalian cells contain a millimolar concentration of PAs [4]. In 1678, the SPE was first identified by Van Leeuwenhoek as crystals in dried semen but not in fresh ones. In 1791, Vauquelin identified these crystals as an unknown phosphate-derived compound [5]. Further, Schreiner reported SPE as a basic compound in 1878, while Ladenburg and Abel proposed its name “spermine” in 1888 [6,7]. After one decade (1898), Poehl suggested the use of SPE for the treatment of several diseases [8], and finally, in 1924, SPE, SPD, and PUT were synthesized by Rosenheim, which led to the foundation of the modern science of PAs [9]. Moreover, the PUT was discovered in the microorganisms in ~1800s, and SPD was identified in the 20th century [10].

PAs have been found to be involved in various important biochemical roles, such as synthesis, functioning, maintenance, and stability of nucleic acids (DNA and RNA), and proteins [11]. They also play a pivotal role in cell signaling, DNA binding, transcription, RNA splicing, and functioning of cytoskeletons, and Eukaryotic translation by maturing translation initiation factor 5A (eIF5A) [12,13,14,15,16]. The numbers of positively charged amino groups linked with each PA are the key factors behind the activity of PAs. The higher positive charge denotes the higher interacting activity with cellular ions [17]. For instance, SPE is a highly active PA because of its four positively charged amino groups, while PUT shows the lowest interacting activity since it contains two amino groups [18]. Therefore, PAs regulate the electronic equilibrium, electric excitation, and cardiac activity by facilitating K^+^ movement into K^+^ (Kir) channels of different cell types [19,20]. They also control connexins and transient receptor potential cation (TRPC) for contractility and excitability of gastrointestinal smooth muscle [4]. Genomic studies showed that PAs regulate the cellular metabolic pathways, which consequently facilitate the formation of subcellular compartments of cytoplasm, mitochondria, and nucleus [2]. Biologically, PAs and their analogues possess functional involvements in human health and diseases, such as gastroenterology [21], oncology [22,23], oxidative stress, cerebral strokes [24], parasitology [25], apoptosis [26,27], obesity [28], asthma [29], and other ailments [2]. SPE and SPD work as the substrates for different biological enzymes to form cytotoxic metabolites by the activity of spermine oxidase (SMO), monoamine oxidase (MAO), copper amine oxidase (CuAOs), and polyamine oxidases (PAO) [30,31]. Amine oxidases (ASOs) are the key molecules behind the regulation of Mono, di, N-acyl amines, and PAs. PAOs produce toxic metabolites such as aldehyde(s) and H_2_O_2_ by oxidative deamination of PAs and biogenic amines. H_2_O_2_ and aldehyde(s) cross the inner mitochondrial membrane and react with endogenous structures and molecules in order to induce the death of tumor cells [32]. In vitro cytotoxicity can be induced in the presence of internal PAs or external SPE in various tumor cell lines of humans using CuAOs, i.e., bovine serum amine oxidase [31,32]. It can also be performed in vivo using CuAOs injection in the tumor [33].

There are three ways to maintain the PA pool in the body: intestinal microorganisms, de novo biosynthesis (endogenous), and supply through diet (exogenous). These mechanisms simultaneously regulate the synthesis, catabolism, and transport of intracellular PA concentration [34]. However, the exogenous diet provides the maximum quantity of PAs than the process of endogenous biosynthesis. Hence, PAs in nutrition (dietary polyamines) play a crucial role in maintaining the biosynthesis of PAs because distortion in the metabolism of PAs may lead to several health disorders [34]. Various food items contain the required amounts of PAs, i.e., plant-derived foods have mostly PUT and SPD, and meat products mainly contain SPE, while dairy products are rich in SPD and PUT [34]. Several studies have estimated the mean intake value of PAs, and the suggested daily dietary intake of PAs is varied from 250 to 700 µmol. [35,36,37]. A controlled diet, solely or with clinical applications, can be used as an effective treatment against various cancer, cardiovascular diseases, Huntington’s disease, Alzheimer’s disease, and Parkinson’s disease.

Therefore, the present review was compiled to describe the functions, metabolic pathways of PAs, and their effective roles in the prevention of diseases.

## 2. Types, Structures, and Functions of PAs

### 2.1. Types and Structures

**Figure 1:**
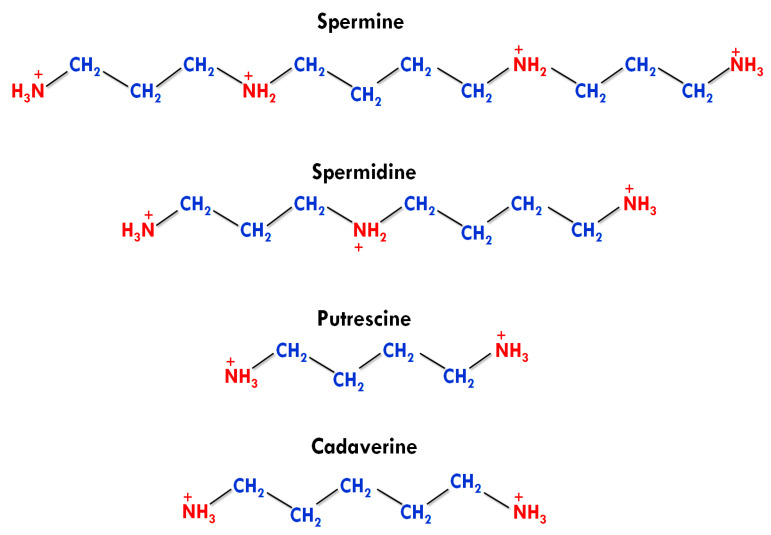
Structures of different polyamines [38].

The native human PAs are PUT, SPD SPE, and cadaverine. Apart from this, agmatine was also detected in human tissues in a trace amount, but it has no active physiological role [4]. The number and presence of amino groups impart different physiological and biochemical roles to biogenic PAs. PUT and cadaverine have two amino groups in their structures and are known as diamines. SPD contains three amino groups and classified as triamine, while having four amino groups, SPE is generally referred to as tetramine (Figure 1) [38].

### 2.2. Functions

**Figure 2:**
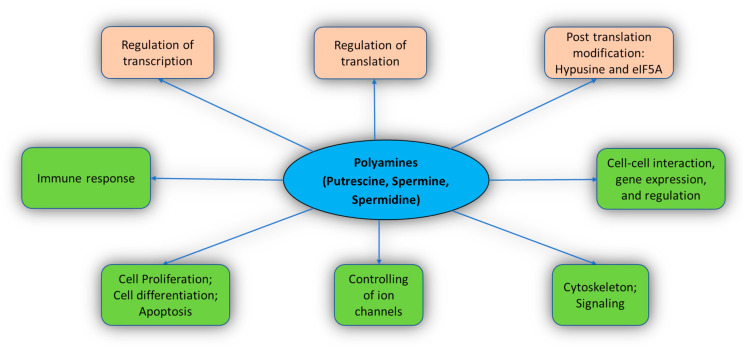
Biological functions related to polyamines.

The functions of PAs include cell differentiation, cell proliferation, gene regulation, cell signaling, and apoptosis [4,18,39,40]. PAs also stimulate post-translation modification with the help of eIF5A (a translation factor) [41]. PAs interact extensively with the cellular molecules and perform various crucial functions in the body (Figure 2). Important known functions of PAs are described below.

#### 2.2.1. Cell Proliferation and Differentiation

PAs are necessary for cell proliferation and differentiation. The rapidly diving cells and regenerative tissues contain a higher amount of PAs [4]. PAs control the expression and stability of p53, a nuclear phosphate protein, which regulates different genes associated with the growth and death of the cell [42]. The depletion of PA by α-difluoromethylornithine (DFMO) leads to an enhancement in the expression of p53, which consequently inhibits cell growth both in vivo and in vitro conditions [17]. SPD was found to be effective in endothelial injury. It helps in the migration of vascular smooth muscle cells after endothelial injury [43]. Various cell-cultured studies confirmed that a higher SPD and lower SPE levels may maintain the normal growth of the rodent cells [44,45]. When cultured cells were treated with an ODC inhibitor such as DFMO, it depleted both SPE and SPD and inhibited viability and proliferation. Moreover, it also enhanced apoptosis [46]. In addition, bis(ethyl)norspermine (BENSPM) was used as an analog of SPE, and it was observed that BENSPM depleted PUT, SPD, and SPE by enabling the polyamine catabolic enzymes, such as spermine oxidase and spermidine N1-acetyltransferase. It consequently halted cell growth [29,47].

#### 2.2.2. Gene Expression and Regulation

PAs are highly positive molecules that bind on the acidic sites of different macromolecules, such as nucleic acids, proteins, and membrane phospholipids [39]. PAs govern various gene expression activities and their regulation as well [17,39]. A possible interaction has been observed between PAs and nucleic acids. For instance, the expression of ornithine decarboxylase antizyme (ODA) is controlled by PAs via the transitional frameshifting mechanism, i.e., the ODA transcript, shows two sequences named ORF1 and ORF2. These ORFs partially overlap, and to correct the overlapping, the PAs create a shift in the messenger reading frame with the help of ribosomes, which further rectify the translation of the second sequence [48]. Moreover, PAs’ interaction with RNA has also been illustrated in the presence of physiological Mg^2+^ ions [41]. PAs act at different levels during protein expression, i.e., initiation of 30S subunit of the ribosome for assembly, protein expression at the cellular level, and initiation to form Ile-ANRt [49,50].

The transcription of many genes, including c-Jun and c-Myc, are regulated by PAs [39]. Likewise, selective PAs are responsible for the regulation of AdoMetDC, AZ, and SSAT for the translation of various mRNA sections [51,52,53]. Additionally, several studies showed the effect of PAs on the cell signaling pathways by affecting the status and levels of main regulatory proteins such as CDK-4, GSK-3β, p53, p27Kip, p21Cip1, Src, EGFR, Mdm2, Akt/protein kinase B, and importin-α1 [54,55,56,57].

#### 2.2.3. Transcription, Translation, and Post-Translation (Hypusine and eIF5A)

The interaction of PAs with RNA affects the level of individual cell proteins in several ways such as facilitating initiation complexes formation, change in the structures of ribosomes, and enhancing frameshifting [58,59]. PAs can also influence the protein structure by the direct or indirect effect on the degradation and processing of post-translational protein [60,61].

During post-translation modification, SPD donates the aminobutyl group to the translation factor (eIF5A) with the help of deoxyhypusine synthase enzyme, which consequently results in the formation of hypusine (*N^ϵ^*-(4-amino-2-hydroxybutyl) lysine) [62]. This is an important modification step for the activity of eIF5A because it may help in nucleocytoplasmic transport, transcription, mRNA turnover, and apoptosis [14,16,63,64], but eIF5A is best known for the translation of polyproline stretches of mRNA, i.e., PP*X* (*X* represents Asp, Asn, Gly, or Try) [65,66]. These stretches act as the binding sites for ribosomes. After binding, hypusinylated-eIF5A moves toward the ribosome’s peptidyltransferase point to orient and stabilize the CCA part of the peptidyl-RNA for further translation [67]. Proteins having these proline stretches regulate several functions, such as DNA binding, transcription, RNA splicing, cell signaling, and cytoskeleton-related functions for the development and growth of the cells [16,68]. Vertebrates possess another gene encoding eIF5A2, which expresses less and is not crucial for the body; however, eIF5A2 has been found in various cancer cells, responsible for poor prognosis and rapid growth [14,16]. It was observed that tumor growth and the expression of oncogenic tyrosine kinase (PEAK1) can be inhibited by preventing the formation of hypusine in eIF5A2 [69]. According to research, polyamine reduction activates the phosphorylation of eIF2α (translation initiation factor) and PERK (stress-responsive kinase), which shows an insightful role of PAs in the initiation of translation [70]. Moreover, PAs’ depletion in mammalian cells using an inhibitor of SPE synthase, i.e., difluoromethylornithine plus *N*^1^-(3-aminopropyl)-cyclohexylamine showed an inhibitory effect on cell growth by impacting hypusine level [41].

#### 2.2.4. Regulating the Function of Ion Channels

##### Inward Rectifier Potassium (Kir) Channels

Kir channels represent a superfamily of K^+^ ion channels, such as voltage-gated, two-pore, cyclic nucleotide-gated, and calcium-gated channels [4]. The potassium flux via Kir channels maintains the electrolyte equilibrium, membrane potential, and electron activity of neurons and cardiac muscles. A study of *Xenopus* oocytes revealed that polyamine binding initiated the rectification in the HRK1 Kir channel, which was observed subsequently in the large family of such Kir channels [71]. This brought a small change in the concentration of PAs because of the higher potency of SPE than SPD, which was responsible for a significant change in the activity of Kir channels [71,72].

In a structural study of Kir1 to Kir7 subfamilies, it was observed that the PAs first bind to cytoplasmic pore at a shallow binding site with low voltage dependence and then move toward a deep position through a long pore. This position is called the rectification controller or acidic residue, which interacts with PAs to initiate steep voltage dependence [73,74].

##### Transient Receptor Potential Canonical (TRPC) Channels and Connexins

TRPC channels are comprised of a seven-member family (TRPC-1, 2, 3, 4, 5, 6, and 7) in the mammalian cells. They are nonselective cationic and calcium-permeable channels, which primarily work at the plasma membrane [75]. Additionally, they act as second messenger-operated and store-operated channels, responsible for contractility and extractability of smooth gastrointestinal muscle [4]. Intracellular PAs, specifically SPE, interact with two glutamate residues and inhibit TRPC-4 and TRPC-5 [76]. On the other hand, intracellular SPE increases the communication between astrocytes and also in gap junctions [77]. It also helps in coupling connexin Cx43 channels at low pH [78].

##### Ligand-Gated Ion Channels

Synaptic plasticity and synaptic transmission that determine learning and memory occur in the cellular membrane by the binding of glutamate (ligand). It is a part of the inotropic glutamate receptors family [79]. There are three classes of these receptors and each class has many members on the basis of their active agents, such as AMPA, NMDA, and kainate. PAs can influence the activities of the members of these classes [4]. Few NMDA receptors work as voltage-dependent and ligand-gated channels to control synaptic plasticity [80,81]. PA effects include inhibition and stimulation of a voltage-dependent channel, which depicts an open-channel block. In addition, PAs facilitate the binding of NMDA receptors on the extracellular sites of these ion channels [82,83]. SPE has been found comparatively potent than SPD for these effects [4].

PAs also impact the AMPA receptors family, which do not have glutamate subunits [84]. AMPA receptors act as neurotransmitters to regulate synaptic power and enhancing neurotransmission in the central nervous system (CNS). Intracellular PAs, potently SPE, have the capacity to block these channels, which bind on the pore region of the channels [85]. Notably, PAs can regulate the excitability limit of synapses and the concentration of Ca^2+^ flux.

#### 2.2.5. Immune Response

PAs have important roles in the immune response. It has been reported that autoreactive B cells and T cells along with cancerous cells contain a higher concentration of PAs during autoimmune diseases [12]. The L-arginine catabolism in suppressive myeloid and tumor cells decreases the functions of cytotoxic T cell, which suggests a link between T cell suppression and PAs [12]. It has been observed that the higher concentration of PAs in an autoimmune patient form a nuclear cluster that reacts with RNA, DNA, and other molecules for stabilizing autoantigens [86]. The formation of single-stranded or double-stranded DNA is the predominant response of autoimmune B cell [87].

#### 2.2.6. Regulation of Transglutaminase

Transglutaminases (TGases) are ubiquitous calcium-dependent enzymes, which perform several cell functions. TGase was first identified in the liver at the incorporation time of amines into proteins [88]. As per the mechanism, a thioester intermediate (acyl-enzyme) interacts with a proper nucleophile after its formation between the polypeptide-bound glutamine and cysteine active site [89]. PAs were reported to regulate the activity of TGase in many functions, including cell differentiation, post-translational protein modification, kinase activity, wound healing, and signal transduction [88,89,90]. Mammalian tissue transglutaminase (TG2) catalyzes protein post-translational change by adding PAs into protein or forming epsilon lysine bonds in an inter- or intramolecular cross-link manner [91,92]. On the other hand, a higher enzyme activity of TG2 was found to be associated with various neuropathological conditions (acute and chronic) such as amyotrophic lateral sclerosis (ALS), Huntington’s disease, Alzheimer’s disease, and Parkinson’s disease [93,94]. It was observed that the actions of superoxide dismutase and cytochrome *c* oxidase decreased with the increased activity of TGase, which consequently leads to dysfunction of motor neurons in the ALS animal model [95]. It has been observed that the activation of glia leads to these neuropathological disorders due to oxidative stress. For instance, primary astrocytes (cultured) were exposed to glutamate (excitotoxic) that led to oxidative stress with TG2 up-regulation. Further, glutamate-induced impairment resulted in the increment of intercellular reactive oxygen species (ROS) and the depletion of glutathione (GSH) [96]. Inversely, pretreatment of astrocytes with antioxidants such as cysteamine-HCL, genistein, GSH ethyl ester, and IRFI-016 reversed the glutamate induced-effect and decreased the level of TG2 [96].

In a study, the activation of nuclear factor-κB was found to be involved in the development of ROS and the activation of TG2 upregulation when the cultured astrocytes of rat hippocampus were exposed to lipopolysaccharide (LP) [97]. LP is commonly used to stimulate iNOS induction. They reported a suppressed level of LP-induced effect after the treatment of ammonium pyrrolidine-1-carbodithioate (nuclear factor-κB inhibitor) in the astrocytes [97].

## 3. Metabolic and Transport Pathway of Polyamines in Humans

**Figure 3:**
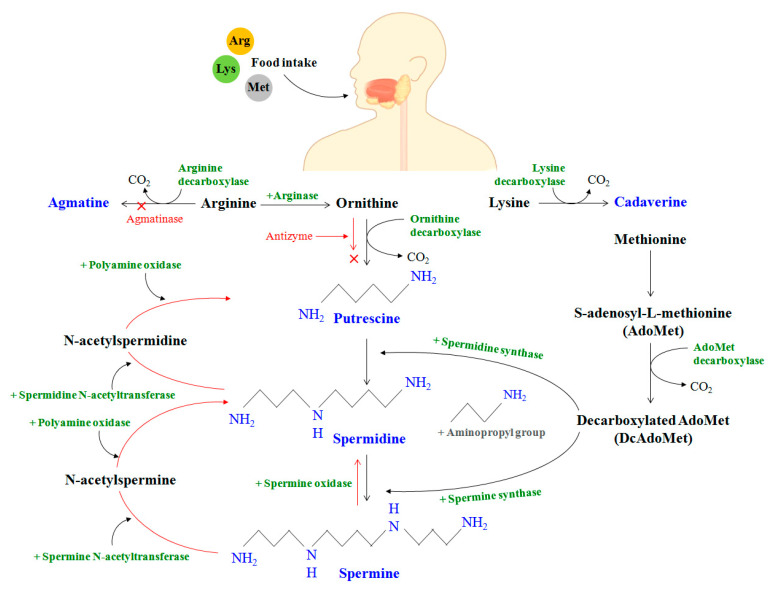
Polyamine synthesis (black/blue) and regulatory (red) pathways in the human gut after ingestion of amino acids: arginine (Arg), lysine (Lys), and methionine (Met).

The homeostasis of PAs in the mammalian species can be understood through three steps that can be broadly classified as synthesis, catabolism, and transport. PAs are produced in the cell cytoplasm. In vivo production of polyamine begins with the intake of amino acids (arginine, lysine, and methionine) through food, serving as substrates for polyamine synthesis through the action of micro-organisms/enzymes [2] (Figure 3).

In the mammalian gut, the enzyme arginase first decomposes the amino acid arginine to produce ornithine. Ornithine is also generated as a product of the urea cycle [98]. The accumulated ornithine is then decarboxylated by the action of the ornithine decarboxylase (ODC) enzyme to produce the polyamine, PUT. Meanwhile, methionine is transformed to S-adenosyl-L-methionine (AdoMet), which is further converted to decarboxylated AdoMet or DcAdoMet in the presence of the AdoMet decarboxylase enzyme. The DcAdoMet thus produced serves as an aminopropyl group donor to putrescine for the synthesis of spermidine in the presence of spermidine synthase. DcAdoMet can also serve as a donor to spermidine for the synthesis of SPE in the presence of spermine synthase. It should be noted that both ODC and AdoMet decarboxylase are PA-rate-limiting enzymes that are strictly controlled at the transcriptional and post-transcriptional stages.

Though the formation of PAs in mammals is important to nucleic acid stabilization and, cell growth and proliferation, excess of PAs can be toxic and can cause skin cancer, colon cancer, and increased oxidative stress due to the formation of abnormal cells and peroxides [17]. Therefore, the regulation of PAs should be actively handled within the cells. In this regard, the production of SPE and SPD is regulated through an interconversion pathway wherein they can be acetylated and oxidized back to putrescine marking the stage of catabolism [39]. Acetylation reduces the interaction of PAs with polyanions reducing their positive charge. The cytosolic spermidine/spermine N-acetyltransferase (SSAT) and polyamine oxidase (PAO) are jointly responsible for the mechanism of polyamine catabolism. PAO is more actively involved in spermine catabolism than spermidine. A higher degree of regulation involves the action of other oxidases, along with their cofactors, within the body to generate permanently polyamine derivatives from amino acids that cannot be recycled back to PAs. Another regulatory mechanism involves the ubiquitin-independent degradation of ODC by antizyme (AZ), thereby arresting the production of PUT altogether. In contrast to the antizyme-based regulatory mechanism, an antizyme inhibitor (AZIn) enzyme can rescue ODC from rapid degradation due to its higher binding affinity to Az than to ODC [99,100]. This is because AZIn is homologous to ODC despite lacking enzymatic activity [101]. AZIn can therefore bind to AZ, releasing ODC in the process that accelerates polyamine formation. Apart from PUT, SPD, and SPE, other PAs can also be synthesized within the body. For instance, the polyamine agmatine, which acts as a neurotransmitter, can be produced by the decarboxylation of arginine; however, it is instantly degraded due to the presence of an active enzyme agmatinase in the human gut [39]. Although agmatine is readily degraded, studies have shown the presence of trace agmatine in selective regions of the human brain and other human tissues [102]. It was established that the human agmatine decarboxylase (ADC) enzyme represents a 460-amino acid protein and is 48% similar to the human ODC but with no ODC activity. The metabolism and function of agmatine in the human body are still not fully explored and require ADC gene characterization and intensive regulatory investigations to develop a complete understanding. Cadaverine, synthesized from decarboxylated lysine, is yet another polyamine that has structural similarity to PUT and is produced in the presence of lysine decarboxylase [103]. Both PUT and cadaverine have a pungent smell and are related to cellular decomposition due to which they are often called “necromones,” indicating cell death [104]. Polyamine synthesis and degradation can also be affected by aging. For instance, it has been shown that the formation of PUT is positively correlated to aging, while it is negatively correlated with SPE and has no significant correlation with spermidine [40]. It was concocted that the increase in spermine oxidase expression with age could cause oxidative degradation in the levels of spermine. Moreover, due to basal levels of SSAT and PAO, spermidine can be transformed into putrescine. The extent of conversion is, however, affected by age, and hence, the polyamine profiles are significantly altered in the elderly.

Polyamine regulation in the body is also facilitated by polyamine transport and cellular uptake. PA transport is known to be mediated by solute carrier (SLC) and ATP-binding cassette (ABC) transporters. SLC3A2 and SLC22A16 are the more popularly known PA transporters [98]. In a later investigation, Abdulhussein and Wallace [105] studied the PA transport mediating potential of ABC and eight SLC transporters (SLC22A1, SLC22A2, SLC22A3, SLC47A1, SLC7A1, SLC3A2, SLC12A8A, SLC22A16). They reported that the MDR1 protein of the ABC superfamily could mediate PA-like molecules, while SLC22A1 may aid in PA uptake. Hamouda et al. also reported an unexplored gene, ATP13A3, as a potential candidate for PA transport that complemented PUT transport deficiency [106]. In another recent study, the gene SLC18B1 of the vesicular amine transporter family was identified as a transporter of spermine and spermidine. Knockdown of the SLC18B1 gene showed a 20% reduction of PA in the brain, which was said to adversely affect short- and long-term memory [107].

The polyamine transport system (PTS) requires energy, is concentration, time, and temperature dependent, and is saturable [108]. The gut-bacteria-derived PAs are transported into the bloodstream via the colonic mucosa [109]. The PTS can, however, be effectively harnessed for targeting specific cells, which opens up a broad spectrum of medical applications. It has been demonstrated that cancer cell proliferations have high polyamine transport activity, and the transport system holds relevance as a target site for selective drug delivery. Taking advantage of the intrinsic needs of cancer cells to utilize polyamine metabolites for growth, Muth et al. [110] showed that the novel compound *N*^1^, *N*^1^-Naphthalene-1,4-diylbis(methylene)]bis{N4-4-(methylamino)butyl])butane-1,4-diamine}, 3b, had excellent polyamine transport system selectivity and was stable to amine oxidases, making it a candidate for targeting breast cancer cells and melanomas. Polyamine transport has also been investigated in colorectal cancer cells [111] wherein the polyamine transporter was exploited to be used as a potential anticancer drug carrier. It was established that the rates of cell growth and polyamine depletion were associated with polyamine transport. It was also observed that the attenuation of PAs invigorated the transporter affinity for PUT and not for the long-chain polyamine, SPD. In another investigation, polyspecific organic cation transporters (OCTs) were explored for potential binding and transport of longer chain PAs [112]. It was shown that SPD uptake rates increased by threefolds, compared to noninjected oocytes. Overall, the PTS is less explored and more focus needs to be diverted toward identifying novel disease-specific PA transporters.

## 4. Nutritional Roles of Polyamines in Health Maintenance and Disease Prevention

### 4.1. Aging and Longevity

Aging is a complex process defined differently by various researchers. It has been perceived by Denham Harman that progressive changes accumulate in the body with the passage of time, increasing the possibility of the development of diseases or death of the individual [113]. As an organism ages, the levels of PAs decrease [114]. Various organs in aging humans including serum and other aging mammalian cell cultures have shown a positive correlation with reduced levels of intracellular PAs [115]. This has encouraged researchers to study the effect of polyamine supplementation on longevity. The 24-week old Jc1:ICR male mice fed with spermine and spermidine at levels ranging from 143 nmol/g and 224 nmol/g to 374 nmol/g and 1540 nmol/g, respectively, showed increased levels of these PAs in whole blood with a significant increase in spermine levels. The consumption of these two PAs by the mice also increased their life span with a more prominent effect shown at higher doses (*p* = 0.011), as compared to moderate and low doses. Eisenberg et al. [115] studied the role of spermidine in inducing autophagy and suppressing necrosis, key factors that promote longevity in organisms such as yeasts, flies, worms, mice, as well as human cells. Aging wild-type BY4741 yeast cells and DB4746 cells showed increased life span after spermidine treatment with a four-time increase in the life span of wild-type BY4741 cells, as compared to the control group of cells with an increase in intracellular spermidine levels. Similar results were observed for the increase in the life span of fruit fly *Drosophila melanogaster* (30% increase) and nematode *Caenorhabditis elegans* (15%) after administration of 1 mM and 0.2 mM spermidine, respectively.

In addition to amelioration of chronological aging, spermidine administration rejuvenates replicative old cells. Spermidine administration (20 mM for 12 days) improved the survival of human peripheral blood mononuclear cells (PBMCs) cultures by 50%, as compared to survival of only 15% of cells in control cultures, by inhibiting necrosis as spermidine reduced the cell death associated with membrane rupture and deacetylation of histone [115]. Moreover, spermidine upregulated autophagy-related genes such as *ATG7*, *ATG11*, and *ATG15* and significantly increased specific hyperacetylation of the promoter region of *ATG7* (*pATG7*), maintaining the accessibility of the promoter region and thus allowing for its transcription as evident during chronological aging of yeast [115].

Likewise, lower levels of TFEB, hypusinated eiF5A, and autophagic flux is reported in defective B cells obtained from humans ≥ 68 years of age, which were restored to the levels seen in young B cells after spermidine treatment [116]. Hypusinated eiF5A is the only protein that contains hypusine amino acid generated from the conjugation of spermidine aminobutyl moiety and acts as an elongation factor for translation of polyproline by peptide bond formation [66], is required by transcription factors such as TFEB and TFE3 for translation. TFEB and TFE3 are further involved in the transcription of coordinated lysosome expression and regulation (CLEAR) of genes responsible for lysosomal biosynthesis and for encoding autophagy-related proteins [117]. Puleston et al. [118] documented the role of eiF5A in regulating mitochondrial localizing sequence containing nuclear-encoded mitochondrial proteins translation. In addition to life span extension, polyamine supplementation reduced the age-associated rise in proinflammatory status and pathological changes. Spermine treatment further improved DNA methyltransferase activity, improving altered DNA methylation status in HT-29 and Jurkat cells [119].

There are several theories behind the process of aging of which the free radical theory is most prominent. According to this theory, as an organism ages, oxidative stress accumulates in the body due to the formation of free radicals during metabolic processes. This accumulation of oxidative stress contributes to a reduced life span as it plays a cardinal role in the development of various degenerative metabolic diseases. Eisenberg et al. [115] demonstrated a 30% increase in serum levels of free thiols in C57BL/6 mice treated with 3 mM spermidine for 200 days, thus reducing age-associated oxidative stress.

Further, elevated proinflammatory status promotes the process of aging since it leads to the development of many age-related chronic diseases. An improvement in longevity by a reduction in proinflammatory response can be achieved by inhibiting the binding of intercellular adhesion molecules (ICAMs) and lymphocyte function-associated antigen 1 (LFA-1), which, in turn, produce inflammatory responses [120,121]. LFA-1 is composed of CD11a (an alpha-L chain) and CD18 (a beta-2 chain). Thus, inhibiting the LFA-1 function or downregulating the binding of ICAM and LFA-1 can retard inflammation and consequently lead to aging. Flow cytometry analysis showed that treatment of human PBMCs obtained from healthy volunteers with spermine for 72 h suppressed the CD11a and CD18 expression, as observed from their reduced mean fluorescent intensities (MFIs) [122]. This inhibitory effect of spermine on the expression of CD11a was further confirmed by supplementing the D,L-alpha-difluoromethylornithine hydrochloride (DFMO) (3 mM) treated Jurkat cells with spermine (500 µM), which had significantly decreased the expression of CD11a to 94.87% ± 3.93%, compared to only DFMO-treated cells, while increasing the methylation of LFA-1 gene (ITGAL) promoter area. DFMO selectively inhibits ornithine decarboxylase, an enzyme responsible for polyamine synthesis, thus creating a polyamine deficient state. Additionally, the MFIs of CD11a were negatively associated with DNA methyltransferase (Dnmt) activity. Dnmt is an enzyme responsible for methylation of cytosine by taking methyl group from S-adenosylmethionine (SAM) [123]. The presence of methylated cytosine at the transcription site of the gene can suppress the process of aging if that gene codes for age-related diseases. Similarly, demethylation of the genes responsible for suppressing age-related diseases will be helpful in reducing the aging process [124].

### 4.2. Stress

The role of PAs in improving the life span in yeast, worms, flies, mice, and in cell cultures of human are well documented by Eisenberg et al. [115]. Additionally, improved longevity is often strongly correlated with increased stress resistance [125]. On the other hand, as the person ages, the generation of oxidative stress from the formation of free radicals, a result of various metabolic processes, also increases inducing the risk of various age-associated degenerative diseases. Thus, the beneficial role of PAs in reducing oxidative stress and stress generated from starvation has been explored by Minois et al. [126] in their experiments in *Drosophila melanogaster* (fruit fly).

The spermidine treatment (0.1 mM) to male and female fruit flies pretreated with 5 mM paraquat improved their climbing on the vial vertical wall in which they were kept by approximately 30% and survival rate by enhancing autophagy. Paraquat is used by researchers as a neurotoxic agent to develop neurodegenerative disease associated with age since it generates superoxide anion in *D. melanogaster* [127]. Eisenberg et al. [115] reported increased resistance to hydrogen peroxide (H_2_O_2_) and heat-shock-induced stress in spermidine treated yeast cells. Similar results were observed by Minois et al. [126] in fruit flies exposed to 1% H_2_O_2_.

Alternatively, low levels of PAs increased stress levels in animal models. For instance, polyamine catabolism in spermidine/spermine N(1)-acetyltransferase (SSAT) overexpressing transgenic mice had increased H_2_O_2_ production, coupled with 23% and 42% reduction in Cu, Zn-superoxide dismutase, and catalase levels, respectively, and a 60% decrease in CYP450 2E1 expression. These metabolic changes further elevated the level of oxidative stress, as evident from a tenfold increase in protein carbonyl content and overexpression with hepatic transcription factor p53 with a 50% reduction in the life span of these mice [128].

### 4.3. Memory

The role of PAs, i.e., spermidine, putrescine, and spermine, in learning, memory, cell proliferation, neuroprotection, and neural differentiation has been studied by various researchers [129,130,131]. *Drosophila*, an ideal model for studying age-associated memory impairment (AMI) due to its shorter life span, coupled with advanced genetic, was used by Gupta et al. [132] to study the effect of polyamine consumption on AMI. In the study, feeding spermidine (1 mM and 5 mM) to isogenized wild-type flies improved both short-term and intermediate-term olfactory memory performance scores in 30 days old flies, comparable to young flies. Interestingly, the restoration of memory occurred when PAs (both spermine and putrescine fed individually) were fed for a period of 10 days immediately before conducting the memory test (on the 30th day of their life), while this was not observed when PAs were fed for the initial 20 days of life and withdrawn for 10 days before the memory test. This amelioration in AMI by spermidine may involve several parallel pathways along with induction of autophagy [132]. Fabbrin et al. [133] reported an improvement in fear memory consolidation post spermidine treatment (2 nmol/site) given immediately after training in adult male Wistar rats, whereas ANA-12, a TrkB antagonist, inhibited the positive effect of spermidine on memory consolidation. Signor et al. [134], in a series of experiments on rats to study the effect of spermidine consumption on fear memory reconsolidation and neural differentiation, noted that reconsolidated memory persistence increased in a time-dependent manner when spermidine is administered (intrahippocampal infusion (i.h.)) immediately (*p* = 0.005) or 12 h (*p* = 0.007) post reactivation session (*p* = 0.005), as the freezing score increased to ~80% and ~65%, respectively, as compared to ~40% in the control group during the testing session conducted 7 days post reactivation. However, in the absence of a reactivation session, the contextual fear conditioning remained unaltered by spermidine administration. The role of spermidine in improving the contextual fear memory when it is administered 30 min before training, immediately after training, or immediately after reactivation has been reported by various researchers [135,136,137,138]. The increase in the persistence of reconsolidated memory is because of increased levels of mature brain-derived neurotrophic factor (BDNF) in the hippocampus of the Wistar rats post spermidine (2nmol/site) treatment, while the levels of total BDNF remain unaltered. BDNF is a neurotrophic factor abundantly present in the cerebral cortex and hippocampus of the adult brain, playing an important role in memory formation and retrieval [139]. Similarly, spermine reverses the memory impairment caused by lipopolysaccharide (LPS) by improving BDNF levels and activating tropomyosin-related kinase B (TrkB) receptors [140]. Mature BDNFs are known to bind with TrkB receptors, thus strengthening synaptic plasticity and transmission [141]. In addition to TrkB, Fabbrin et al. [133] documented the involvement of phosphatidylinositol 3-kinase (PI3K)/Akt pathway in facilitating spermidine to induce memory consolidation as PI3K inhibition prevents spermidine induced Akt phosphorylation, thereby impairing consolidation and acquisition of both short-term and long-term memory [142]. Phosphorylated Akt plays an important role in the memory formation process since its concentration increase 10–40 min after learning [143,144].

The administration of SPE (0.3 mg/kg b.w.) to swiss albino male mice preadministered with saline or LPS (250 µg/kg b.w.) restored the levels of mature BDNF in both hippocampi (to ~300 pg/mL in LPS–spermine-treated group, compared to ~300 pg/mL in saline–saline group) and cerebral cortex (to ~340 pg/mL in LPS–spermine-treated group, compared to ~350 pg/mL in saline–saline group) and total BDNF in the hippocampus (to ~1200 pg/mL in LPS–spermine treated group, as compared to ~900 pg/mL in saline–saline group) otherwise reduced by LPS treatment. The improvement in BDNF levels was the result of an increase in phospho-cyclic AMP (cAMP)-responsive element-binding protein (CREB) immunoreactivity and phospho-CREB/total-CREB ratio in LPS-treated cerebral cortex of mice [140]. CREB is a transcription factor that enhances memory consolidation [145,146] by increasing the expression of BDNF [147], while its active form, i.e., phosphorylated CREB, promotes the transcription of memory-associated genes [148].

Additionally, in vitro studies revealed that as a consequence of spermidine treatment (10 nM), the migration of neurons increased on day 1 of differentiation, while neurites count increased on day 7 of differentiation of neural progenitor cells (NPCs) without affecting their length. Signor et al. [134] reported the involvement of GluN2B-containing *N*-methyl-D-aspartate (NMDA) receptors, protein synthesis, and role of protein kinase A (PKA) pathway in increasing persistence of fear memory when spermidine is administered (i.h.) 12 h after training. Moreover, Guerra et al. [149,150] documented the involvement of protein kinase A/CREB and protein kinase C signaling in rats hippocampus for memory consolidation induced by spermidine treatment, indicating the positive effect of PAs in memory consolidation.

### 4.4. Cardioprotective Role

The risk of cardiovascular diseases increases as the person ages since aging can lead to stiffness of large elastic arteries as well as the development of vascular endothelial dysfunction [151,152]. Cardiac aging is a result of altered protein homeostasis caused by oxidative stress, leading to vascular dysfunction [153,154,155]. A natural phenomenon of recycling damaged biomolecules known as autophagy plays a cardinal role in the prevention of cardiovascular diseases through preventing or reversing age-associated arterial dysfunction [156].

Spermidine supplementation has proved to be beneficial in cardiovascular diseases by promoting autophagy. In old male C57BL6 mice (27–29 months with approximately 50% survival rate), 3 mM spermidine administration via drinking water for 4 weeks increased expression of aortic LC3-II, an autophagy marker, along with reduction of p62, a marker of undegraded autophagy substrate, which was otherwise altered with aging in mice, as compared to young (4–6 months) control samples. Similar results were observed by Eisenberg et al. [157] with an enhanced mitophagy and improved structure and function of cardiomyocytes in the spermidine-treated group of mice. The beneficial effect of spermidine on autophagy was mediated by increased Atg3 expression, a core autophagy machinery protein, in suppressed histone H3 acetylation of both old and young mice [156]. Likewise, age-associated hypertrophy detectable by echocardiography was reversed in spermidine administered to old mice, as evident from a reduction in tibia-length-normalized left ventricular mass (LV mass/TL) and posterior wall thickness (PW/TL) to levels lower than those observed in middle-aged WT mice (18 months old) but higher than 4 months old WT mice [157]. Treatment with spermidine later in life significantly enhanced diastolic properties and reduced left ventricular passive stiffness in mice without having much effect on systolic properties, as analyzed through invasive hemodynamic pressure-volume measurements.

LaRocca et al. [156] further added that spermidine administration to old mice alleviated arterial stiffening by decreasing advanced glycation end (AGE) product formation and aortic pulse valve velocity to those comparable to the control group. However, no such effect was observed in young mice. Further, the carotid artery endothelium-dependent dilation (EDD) in response to acetylcholine was normalized after spermidine treatment in older mice, which was otherwise reduced by approximately 25% due to reduced nitric oxide (NO) bioavailability in older mice. Reduced bioavailability of NO, as determined by altered NO-mediated EDD results in vascular endothelial dysfunction [158,159], thus dictating the positive effect of polyamine spermidine administration on cardiovascular health. The arterial endothelial function was again improved as spermidine supplementation reduced the oxidative stress in aortas of both old and young mice, as indicated by the reduction in aortic nitrotyrosine levels and reduced superoxide production in only old mice [156].

The ventricular–vascular coupling (VVC), an indicator of cardiovascular performance, decreases with aging, as was seen in 18- and 24-month-old mice. Spermidine supplementation increased VVC in mice, compared to those in 4-month-old mice; however, it did not cause a change in systemic diastolic and systolic blood pressure. Pulmonary congestion due to increased relative lung weight, as a consequence of abnormal cardiac function, also decreased in the spermidine-treated group, compared to the 24-month-old control group. Further, ultrastructural analysis of old mice hearts conducted by design-based stereology by Eisenberg et al. [157] research group showed increased relative mitochondrial and myofibrillar volumes and reduced sarcoplasmic volumes in spermidine fed group of mice, indicating cardiomyocyte-intrinsic effects of spermidine. It also enhanced the mitochondrial respiratory function and mitochondria-related metabolite levels, which usually decline with aging. The levels of proinflammatory cytokine tumor necrosis factor (TNF-α), which alters cardiomyocytes stiffness [160], were improved post spermidine treatment as it increased the phosphorylation of total and Ser4080 of the N2B isoform of titin [157], which plays an important role in passive stiffness of cardiomyocytes [161].

### 4.5. Cancer Prevention

The controlled diet of PAs can also reduce the growth of tumor cells in cancer patients. In this context, the elimination of intestinal microbiota is necessary without compromising metabolic enzymes along with the regulated supply of exogenous PAs in diet [34]. A diet without PAs increased the efficiency of difluoromethyl ornithine (chemotherapeutic agent) in an animal cancer model, which inhibited ornithine decarboxylase [22]. The decreased level of dietary PAs and intestinal decontamination were found beneficial for the controlling of pain in the patient of prostate cancer [103]. Moreover, tumor progression and oncogenesis were checked by a PA blocker therapy, and an anticancer immune response, along with tumor suppression, was observed by immunosuppression [162].

A study was conducted to investigate the side effects and tolerance of PAs free oral supplementation with 2500-times-reduced-PA diet, and results showed no toxicity, along with a higher tolerance limit. Moreover, gut decontamination was also observed, which consequently provided pain relief to the patient [163]. Other studies also reported a PA-deficient diet as the pain relief treatment [164,165]. Ferrier et al. [166] found that the controlled PA amount was an effective and promising nutritional treatment against acute pain hypersensitivity. PAs ultimately affect the metabolic pathways of the cell to provide relief in acute and severe cancer conditions. As per a study, the hypoplasia of colonic mucosa and small intestine was significantly achieved when a PA-deficient diet was given for a long time period [167]. The dietary PUT decreased the activity of sulindac for suppressing oncogenesis of the intestine in a mouse model, which suggested that the dietary PAs level might be a strategy to prevent colon cancer chemotherapy [168]. On the other hand, a low PA diet reduced the pain and enhanced the health of patients with prostate cancer and colorectal adenoma [169,170]. In a recent study, Huang et al. [171] examined the risk of colorectal cancer associated with dietary intake of total PAs, PUT, and SPD, separately. They found that the higher level of total PAs and a lower level of SPD reduced the risk of colorectal cancer in China.

DFMO is known as an effective therapeutic drug to inhibit ODC because the high expression of ODC has been associated with a high risk of cancer [172]. Hence, DFMO is used as a drug in many cancer patients to target ODC (a PAs synthesis enzyme) for the inhibition of cancer proliferation [173,174]. In a clinical study, DFMO treatment delayed tumor formation in the homozygous mouse, while prevented tumor onsetting in the hemizygous (TH-MYCN) mouse [172]. Similarly, DFMO reduced the level of ODC in MYCN-amplified human neuroblastoma cell lines, which consequently enhanced hypophosphorylation and arrested the cell cycle [175]. A recent study confirmed that the DFMO administration is also an effective drug therapy to treat malignant pleural mesothelioma (MPM) by inhibiting the ODC level [154].

Several studies have shown the DFMO mediated therapy targeting PAs against a different type of cancer; however, human clinical trials are needed on a priority basis for the strong and promising evidence in the form of clinical data. Positively, clinical trials are being carried out to examine the effect of DFMO against bladder cancer, skin cancer, gastric cancer, prostate cancer, oesophageal cancer, and cervical cancer [173].

### 4.6. Huntington’s Disease (HD)

HD is a lethal genetic disorder in which, neurons break down progressively, leading to a memory deficit in the brain. The quinolinic acid was given to the animal model using an intrastriatal injection against HD, and it doubled various neurological and histopathological symptoms, along with a neurofunction loss of HD [176]. Similarly, SPE (10 nmol) dose with an intrastriatal injection weakened the power of object identification in the rodents, while 0.1 nmol dosages of SPE reduced the detrimental effect of quinolinic acid. Moreover, it reduced quinolinic acid-induced astrocytosis [177]. These incidences showed that a higher SPE dose decreases the activity of NMDA receptors, whereas a low dose leads to an increased level of NMDA receptor activity [177]. A study reported an enhanced trauma-induced cognitive deficit in new astrocytes when DFMO was fed in potable water [178]. Similarly, Tunali and Tüfekçi [179] analyzed the effect of DFMO, PUT, SPD, SPE, and cyclohexylamine (CHA) on the mutant huntingtin mediated excitotoxicity of HEK293 cells. They observed that SPD, SPE, DFMO, and CHA increased the levels of mutant huntingtin aggregates. Enhanced viability in huntingtin expressing cells was also reported. These studies showed the regulatory effects of PAs as affected by the different levels of metabolic drugs.

Lipopolysaccharide is a known cell wall component of Gram-negative bacteria that causes memory impairment in the hippocampus and cerebral cortex through neuroinflammation. Lipopolysaccharide-induced nervous inflammation results in learning avoidance, weakened spatial memory, and fear conditioning in rodents [180,181]. The intrastriatal injection with 0.3 mg/kg SPE diminished the lipopolysaccharide-induced inflammation and reversed the memory loss as well [182]. The results revealed that the cognitive deficit caused by lipopolysaccharide is mediated via NMDA receptors [182].

### 4.7. Alzheimer’s Disease and Parkinson’s Disease

In Alzheimer’s disease (AD) and Parkinson’s disease (PD), the cognitive function of the brain degrades gradually with aging. After phosphorylation, *Tau* protein accumulation makes beta-peptide of neurotoxic amyloid (Aβ) and neurofibrillary tangles, which cause various neural ruinations. In addition, it also forms neuritic plaques in the brain [183]. It has been observed that AD patients have a higher level of PAs in the brains, which is found to be associated with synaptic loss and cognitive deficit [184]. Aβ treatment enhanced synaptic loss, NDMA activation, and the level of PAs in the nerve cells culture [185]. NDMA antagonists reversed the effect of cognitive impairment that was induced by Aβ intracerebral injection, which showed memory loss in the test animals [186]. Moreover, Gross et al. [187] found that DFMO and arcaine overturned the induced memory decline effect of Aβ-25–35 by blocking the synthesis of PAs in the mice. These studies confirmed that PAs have a noxious effect against the accumulation of Aβ. PD patients have the suppressed expression of PA catabolic enzyme (SAT1), which consequently elevated the levels of PAs in the patients. This higher PA level reduces the cognitive responses in patients with Parkinson’s disease through the NDMA pathway [188]. Additionally, the accumulation of α-synuclein has also been reported in patients with PD due to a higher level of PAs, but the role of α-synuclein is still unknown [188].

## 5. Conclusions, Current Problems, and Future Perspectives

PAs are the molecules that are synthesized by amino acid decarboxylation and play an important role in several physiological and biochemical processes of living organisms. They control and regulate various important cellular and genetic functions, such as cell proliferation, transcription, translation, and post-translational modifications. It is understood that the functions of PAs depend on the cellular concentration of each PA, i.e., PUT, SPD, and SPE. However, further investigation is needed to understand the homeostasis of PAs in living cells, which facilitates the regulation of biosynthesis, catabolism, conjugation, and interconversion. Moreover, it is also important to know the cellular level of biologically active PAs during stressful condition(s). The dietary intake of PAs revealed that the optimum intake of PAs affects positively by maintaining the health and controlling various diseases. Moreover, PAs slow down the aging process and increase longevity. Various health disorders can also be cured via targeting PAs during the metabolic process.

On the other hand, higher PA levels influence several health disorders such as stress, cancer, and cardio disease. Several studies showed a complex picture of PAs’ effects on different diseases due to their collective use, which provides a gap for future investigations to reveal the role and effect of each PA (PUT, SPE, and SPD) on aging, cancer, memory loss, and Parkinson’s disease. In addition, the dietary intake of PAs showed an alternative path for the treatments of various health disorders. Therefore, the optimized dietary methods can be applied along with clinical applications against fatal diseases for maintaining good health. PAs can be a powerful tool to tackle various health problems if they are tightly regulated for a targeted disease. As a future therapeutic tool, PAs and their analogs may be combined with nanoparticles to formulate the targeted nutraceutical nanodrugs.

## Data Availability

Data have been included within the article. Therefore, no additional data file is required.
